# The Impact of Polyphosphates on the Colloidal Stability of Laponite Particles

**DOI:** 10.1021/acs.jpcb.4c03193

**Published:** 2024-07-09

**Authors:** Bojana Katana, João Baptista, Ricardo Schneider, Rodrigo José de Oliveira, István Szilágyi

**Affiliations:** † MTA-SZTE Momentum Biocolloids Research Group, Department of Physical Chemistry and Materials Science, Interdisciplinary Centre of Excellence, University of Szeged, 6720 Szeged, Hungary; ‡ Group of Polymers and Nanostructures, Federal Technological University of Paraná − UTFPR, 85902-490 Toledo, Paraná, Brazil; § Chemical Engineering, University of São Paulo − USP, 05508-800 São Paulo, Brazil; ∥ Physical Chemistry of Materials Group, State University of Paraíba − UEPB, 58429-500 Campina Grande, Paraíba, Brazil

## Abstract

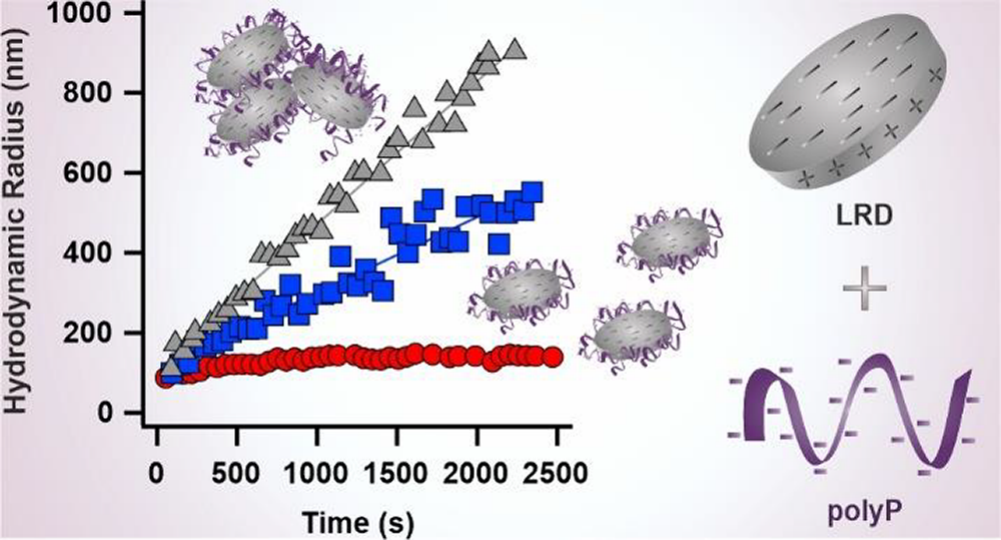

The effect of polyphosphate (polyP) adsorption on the colloidal properties of disc-shaped laponite (LRD) particles was examined in aqueous dispersions with a focus on elucidating the interparticle forces that govern the colloidal stability of the systems. The charge and aggregation rate data of bare LRD exhibited an ionic strength-dependent trend, confirming the presence of double-layer repulsion and van der Waals attraction as major surface interactions. The charge of LRD particles significantly increased in magnitude at elevated polyP concentrations as a result of polyP adsorption and subsequent overcharging of the positively charged sites on the edges of the LRD discs. A transition from stable to unstable LRD colloids was observed with increasing polyP doses indicating the formation of aggregates in the latter systems due to depletion forces and/or bridging interactions induced by dissolved or adsorbed polyP, respectively. The degree of phosphate polymerization influenced neither the charge nor the aggregation mechanism. The findings clearly confirm that polyP adsorption was the driving phenomenon to induce particle aggregation in contrast to other clay types, where phosphate derivatives act as dispersion stabilizing agents. This study provides valuable insights into the early stages of aggregation in colloidal systems involving LRD and polyPs, which have a crucial role in predicting further material properties that are important to designing LRD-polyP composites for applications such as potential phosphate sources in chemical fertilizers.

## Introduction

Laponite (LRD) clays consist of disc-shaped layered particles with magnesium–lithium silicate lamellae of Na^+^_0.7_[Si_8_Mg_5.5_Li_0.3_O_20_(OH)_4_]^−0.7^ composition.^1−3^ The diameter of the unilamellar LRD discs ranges between 25 and 30 nm, while the thickness is about 1 nm.^4−7^ In the crystal structure, an octahedral layer of Mg–O, which is partially substituted with Li–O, is surrounded by two layers of tetrahedral Si–O, and this assembly results in a net negative charge compensated with loosely bound cations,^4^ while the edges are positively charged around neutral pH conditions due to the presence of protonated hydroxyl groups.^2^ Dispersing LRD in water may induce various structural and colloidal transformations, including delamination and the formation of coherent systems from either stable or aggregating dispersions.^1^ The unique morphology of LRD particles provides distinctive interfacial properties that make them different from other clay colloids,^8−10^ and thus, LRD materials are applied in conducting materials, controlled release fertilizers, and drug delivery agents, for instance.^11−13^

The formation of the LRD cluster in dispersion is favored when electrostatic repulsion between the particles is reduced, which can be achieved at high ionic strength or acidic conditions; therefore, adding salts is a suitable tool to reduce the thickness of the electrical double layer (EDL) and to promote the formation of the LRD gels.^14^ In accordance, Joshi^15^ has also reported that LRD particles may form Wigner glass in aqueous dispersions. Mongondry et al.^2^ investigated the influence of the ionic strength on the colloidal stability of LRD and found that the aggregation rate increases with increasing ionic strength. On the other hand, Ruzicka and Zaccarelli^1^ studied the effect of pH on LRD stability and reported individual LRD particles in dispersions at alkaline pH and confirmed that the positive charge at the edges of the LRD particles is attributed to the protonation processes of the surface hydroxyl groups, while the faces possess a net negative charge owing to their structural properties. Furthermore, these charge features influenced the aggregation state of the LRD particles in aqueous samples. Besides, alkaline conditions facilitate the formation of LRD systems with improved rheological properties, including increased viscosity and thixotropy, which are crucial for various industrial and consumer applications.^16^ For instance, LRD is used in cosmetic formulations as stabilizer and thickener, while its stability at higher pH values helps to maintain the consistency and efficacy of lotion, cream, and gel products.^17^

Besides, surface active agents other than salts may influence the colloidal stage of clays. For example, polyphosphates (polyP), an inorganic polymer class, have been demonstrated essential dispersant properties for a variety of colloidal materials.^18−20^ Accordingly, the influence of the length of the polyP chain on the dispersing efficiency in aqueous clay dispersion was revealed by studying different polyP compounds such as Na_5_P_3_O_10_, Na_4_P_2_O_5_, and (NaPO_3_)_6_ with kaolin, illite, and montmorillonite clay particles.^21^

Due to the obvious potential of polyP as dispersant for LRD discs, this topic has received significant attention recently in the scientific community too. Motta et al.^22^ have demonstrated that polyP-bridged particle clusters exhibit repulsive interactions in LRD dispersions. The presence of electrostatic and steric repulsive interparticle forces led to the development of bimodal cluster size distribution due to different LRD aggregation pathways. Mongondry et al.^23^ pointed out that the addition of sodium pyrophosphate inhibits the formation of LRD clusters since the aggregation rate decreases with increasing pyrophosphate concentration. Bujok et al.^24^ investigated the interaction between LRD and phosphate salt constituents such as sodium hexametaphosphate (SHMP), tetrasodium pyrophosphate decahydrate (TSPP), trisodium trimetaphosphate (STMP), and sodium triphosphate (STP). The SHMP and TSPP induced the formation of larger clusters, indicating the presence of attractive forces between the clay particles at certain experimental conditions. In the latter case, it was found that at lower TSPP concentrations, repulsive forces are predominant and they prevent particle aggregation. While increasing the loading of TSPP, attractive forces became more prominent, resulting in the formation of gel-like structures. Besides, STMP and STP generated transparent dispersions, implying strong repulsion between the LRD particles.

It is obvious from the above data that the effect of polyP on the colloidal stability of LRD must be unambiguously understood when such colloids are designed. This requires the knowledge of the intricate relationship between the surface chemistry and aggregation of LRD particles in polyP solutions to utilize these systems in various areas, including tissue engineering,^25,26^ biomedicines,^11,13^ drug delivery,^12^ composite materials,^27^ biosensors,^28^ and the cosmetic industry.^29^ Nevertheless, there is a lack of comprehensive quantitative data on the surface charge and aggregation rate of LRD particles in the presence of polyP.

Therefore, the present study elaborates on the relation between the interfacial processes and colloidal stability in aqueous LRD-polyP samples. Particular attention has been paid to the effect of polyP chain length, concentration, and ionic strength on the charging and aggregation properties of the LRD particles. The results were compared with traditional colloid chemistry theories developed to describe dispersion stabilities in such systems, while the interparticle forces were identified based on the results of light scattering experiments.

## Experimental Section

### Materials

LRD (LAPONITE RD, synthetic modified phyllosilicate, ALTANA, LOT 0002334488, CAS 53320-86-8, bulk density 1000 kg/m^3^, moisture content maximum 10%) in the form of white powder was donated by BYK-Chemie GmbH. Sodium chloride (NaCl) was bought from VWR and used as received. The solutions were prepared with ultrapure water (VWR Puranity TU+). In order to avoid dust contamination, Millex 0.1 μm syringe filters were used to filter both water and salt stock solutions. The measurements were conducted at a temperature of 25 °C, and pH 10 was adjusted with NaOH (VWR). The LRD concentration was maintained at 20 mg/L in the experiments.

### Synthesis and Characterization of polyPs

Condensation during the melting of raw phosphate chemicals provides polyPs with different chain lengths that reflect their characteristics.^30^ For polyP synthesis, 15 g of NaH_2_PO_4_ (Sigma-Aldrich) were placed into a 30 mL Pt/Au crucible. The mixture was melted at a temperature of 700 °C at two different heating rates of 7 °C/min and 20 °C/min during holding times of 60 and 10 min, respectively.

The size of the polyP chains was determined with the classic titration method, as outlined in the study conducted by Momeni et al.^31^ In this method, 0.2 g of the polyP sample was placed in a 0.01 M hydrochloric acid solution. Subsequently, acid–base titration was performed using a 0.1 M sodium hydroxide (NaOH) solution. A microliter pipet was used to add droplets of the solution, which were then stirred and allowed to reach equilibrium before the pH values were recorded. This process was repeated within a pH range of 3 to 11. The process involved a series of the following steps: (i) a first-order equation was derived from the data to represent the relationship between the change in pH and the volume of added NaOH; (ii) specific turning points that indicated transitions in the volume of added NaOH were identified; and (iii) the number of moles of NaOH corresponding to each inflection point was calculated. This allowed the determination of the average molecular weight of the polyP. Finally, the polyP chain size was calculated using the following eq:
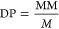
1where DP denotes the degree of polymerization (dimensionless), MM stands for the average molecular weight (g mol^–1^), and *M* represents the molecular weight of polyP monomer (102 g/mol).

The powder X-ray diffraction (PXRD) was performed with a Rigaku SmartLab SE 3 kW (Rigaku) diffractometer equipped with a CuKα X-ray source with a wavelength of 1.5418 Å operating at 40 kV and 30 mA. The diffractograms were obtained by scanning at a rate of 0.05° per minute in the range of 2θ from 5° to 30° in a Bragg–Brentano geometry.

To investigate the elemental composition of polyP, laser-induced breakdown spectroscopy (LIBS) was performed using a J200 spectrometer from Applied Spectra. This device was equipped with a 266 nm laser (25 mJ and pulse width (fwhm) < 6 ns) and a spectrometer with six CCDs with a spectral coverage of 190 to 850 nm and a resolution better than <0.1 nm. The laser power was set to 25%, the gate delay to 0.5 μs, the number of shots to 710, and the spot size to 50 μm. The samples were analyzed directly on the surface of polyP as a tablet, and the spectra obtained were analyzed using the National Institute of Standards and Technology database.^32^

### Electrophoresis

The electrophoretic light scattering technique was used to examine the charging characteristics of the particles with a Litesizer 500 (Anton Paar), which is equipped with a 40 mW semiconductor laser of 658 nm wavelength and operates in backscattering mode at a scattering angle of 175°. The samples consisted of 1.6 mL of polyP solutions of calculated solute and salt contents, as well as 0.4 mL of 100 mg/L stable LRD dispersion, each adjusted to pH 10. The samples were measured using omega-shaped plastic cuvettes (Anton Paar) with a volume of 700 μL. Before recording the electrophoretic mobility, the samples rested for 2 h followed by 1 min equilibration time in the device. The averaged values obtained from five distinct measurements are shown.

To determine the charge density at the slip plane (σ), the electrophoretic mobility (*u*) values were converted to zeta potentials (ζ) using the Smoluchowski model^33,34^ as
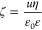
2where ε_0_ refers to the dielectric permittivity of vacuum, ε is the dielectric constant of medium, and η is the dynamic viscosity of water. Thereafter, the surface charge density (σ) value was calculated by fitting the ζ data measured at different salt concentrations with the Debye–Hückel charge-potential linearized relation as^34,35^
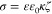
3in which κ represents the inverse Debye length, which depicts the distribution of ionic species in the EDL, i.e., its value is ionic strength-dependent.^33^

### Dynamic Light Scattering (DLS)

In order to get insights into the initial stages of particle aggregation, time-resolved DLS measurements were carried out to determine the aggregation rates. The DLS analysis was conducted with the Litesizer 500 instrument that was utilized for the electrophoresis. The preparation method for the samples was the same as the one outlined for the electrophoretic tests, except that LRD (the final particle load was 20 mg/L in the samples) was added and vortexed for approximately 20 s before DLS measurements. The samples were allowed to equilibrate for 30 s in the device before data recording started. The apparent rate constant (*k*_DLS_) was obtained using the following eq:^36,37,10^
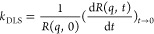
4where *t* is time, *R*(*q*,0) and *R*(*q*, *t*) represent the initial and the subsequent hydrodynamic radii, respectively, while *q* is the scattering vector:
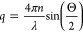
5where λ is the laser light wavelength, *n* refers to the refractive index of water, and θ represents the scattering angle. Note that the scattering vector^38^ is always the same within an identical scattering setup, as it is an instrumental parameter. Besides, the colloidal stability of LRD was expressed in terms of stability ratio (*W*), which is defined as the ratio of the fast aggregation rate coefficient (*k*_DLS_^fast^) and the value measured in the actual experiment:^10,36,39^
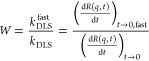
6

Note that *k*_DLS_^fast^ is the aggregation rate during diffusion-controlled aggregation, i.e., when all particle collisions lead to dimer formation and no elastic collision occurs. This can be ascertained at high ionic strengths, where all repulsive electrostatic forces are screened by the dissolved electrolytes.^37^

In general, two regimes can be identified in the stability ratio data, i.e., slow and fast aggregation. Accordingly, the aggregation process becomes diffusion-limited, leading to fast aggregation, when *W* = 1. Higher stability ratios indicate the presence of more stable colloidal dispersions. The limiting value that indicates the power of destabilization is called the critical coagulation concentration (CCC) and represents the salt or aggregation agent dose at which the transition from slow (*W* > 1) to fast aggregation (*W* = 1) regimes occurs:^40^
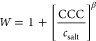
7where *c*_salt_ is the salt (NaCl) concentration (or aggregation agent concentration in other systems), and the exponent β can be obtained from the slow aggregation regime (i.e., before the CCC) from the  versus log*c*_salt_ graphs as follows:^40^
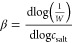
8

## Results and Discussion

### Determination of polyP Chain Size

Distinct levels of acidity can be observed in the hydroxyl groups within the polyP chains depending on their position along the chain. The terminal hydroxyl groups are considered to be weak acids, while the hydroxyl groups in the middle of the chain exhibit stronger acidic properties. The variation in acidity between these hydroxyl groups can be utilized to estimate the average molar mass of polyP.^41^ A titration-based method was developed to determine the average chain length of polyP, which takes advantage of the above-mentioned difference in acidity.

Figure S1 (see Supporting Information) illustrates the relationship between pH and the volume of NaOH added for the two polymers investigated in this study. Two inflection points were observed. The first one was identified around pH 4.5 and attributed to the weak acidic groups. The second point was located at pH 9.0 and referred to the strongly acidic hydroxyl groups. The DP was calculated using eq 1 based on the volume determined between the two inflection points. This value subsequently determines the number of NaOH molecules corresponding to each inflection point to allow the determination of the average molecular weight of polyPs, which were found to be 10542.6 and 15376.5 g/mol. As a result, an average monomer number of 103 (denoted as polyP(103)) was calculated for the higher heating rate and shorter holding time, while 151 (denoted as polyP(151)) for the lower heating rate and longer holding time.

The polyP chain can be classified according to the degree of polymerization as intermediate chain size (DP between 10 and 50) and long chain polyP, when *DP* > 50.^31^ The final chain length depends on the appropriate selection of heating rate, time, and temperature. The DP value obtained for the material matches the degree of polymerization reported in previous studies. For instance, Motta et al.^22^ demonstrated that the use of longer chains is beneficial for more pronounced interaction between polyP and LRD lamellae, as it enhances the attractive interactions between LRD discs.

### Structural Characterization of polyPs

It was reported earlier that phosphate-based glasses can be obtained at relatively low temperatures.^42^ In line with this fact, the PXRD analysis of both polyP(103) and polyP(151) glasses shows the characteristic broad band of the materials in the 2θ range of 15°–30° (Figure S2 in Supporting Information).

Figure S3 (see Supporting Information) shows the LIBS spectra of the phosphate-based glasses obtained with heating rates at 7 °C/min, 20 °C/min, and the NaH_2_PO_4_ precursor chemical. The spectra indicate the presence of the expected elements, such as Na, P, and O. Although condensation during heating results in the release of water molecules, i.e., the elimination of hydrogen, it was assumed that the line at 656.3 nm is present due to moisture absorption.^20^

### Dispersion Properties of LRD

**Figure 1:**
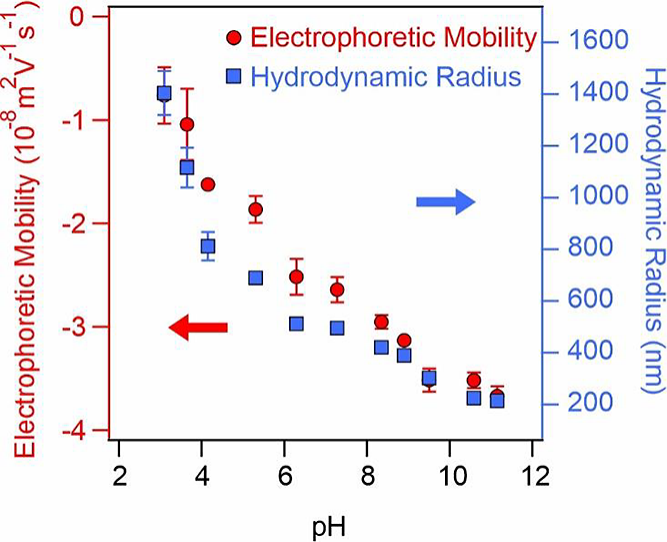
Electrophoretic mobility (red circles, left axis) and hydrodynamic radius (blue squares, right axis) data of LRD as a function of the pH. The measurements were carried out at 1 mM ionic strength due to pH adjustment of the stock dispersions to 3 and 11 and their subsequent mixing. The LRD concentration (20 mg/L) was kept constant in the experiments.

Due to the protonation equilibria of surface functional groups, change in the pH may influence the charging and aggregation properties of LRD particles.^43^Figure 1 shows the pH-dependent electrophoretic mobility and hydrodynamic radius data measured in the pH regime of 3–11.

The permanent structural charge of LRD led to negative electrophoretic mobility values over the entire pH range examined. Furthermore, a continuous decrease was observed in the mobility data upon raising the pH. This tendency can be attributed to the positively charged edges giving rise to less negative mobilities at low pH (−(0.76 ± 0.07) × 10^–8^ m^2^/(V s) at pH 3), while deprotonation progressively occurs and results in more negative charge at higher pH (−(3.67 ± 0.04) × 10^–8^ m^2^/(V s) at pH 11).

The hydrodynamic radii were also determined in the same samples (Figure 1). In line with the above tendency, the hydrodynamic radii decreased with the pH. Accordingly, at low pH, micron-size objects were observed; however, in the alkaline regime, the radius decreased close to 200 nm. Comparing the mobility and radius data, one can notice that the size of LRD decreased with increasing surface charge, suggesting the absence of larger particle aggregates at high pH, at which strong electrostatic repulsion exists between the surfaces.^44^ This can be qualitatively explained by the traditional theory proposed by Derjaguin, Landau, Verwey and Overbeek (DLVO).^34^ Accordingly, in a dispersion of charged particles and electrolytes, particle aggregation is suppressed through EDL repulsion and is caused by permanent van der Waals attractions. The balance of these interactions determines the overall force. Therefore, at alkaline pH samples, highly charged LRD forms stable dispersions due to strong EDL forces,^9^ but in acidic pH samples, in which the particles are weakly charged, van der Waals interactions become predominant and lead to the formation of particle aggregates and, consequently, higher hydrodynamic radii.

Considering these data, the pH 10 condition was chosen for further measurements, at which the electrophoretic mobility was −(3.51 ± 0.10) × 10^–8^ m^2^/(V s) and the hydrodynamic radius was (225 ± 4) nm. The relatively low polydispersity index (0.26 ± 0.01) reflects narrow particle size distribution under this condition. These charge and size data are very similar to the ones reported with hectorite suspensions earlier.^45^ Note that the magnitude of these parameters indicates the absence of larger particle aggregates and allows the use of light scattering techniques to assess colloidal stability of the suspensions.

### Stability Assessment of LRD Dispersions in Salt Solutions

**Figure 2:**
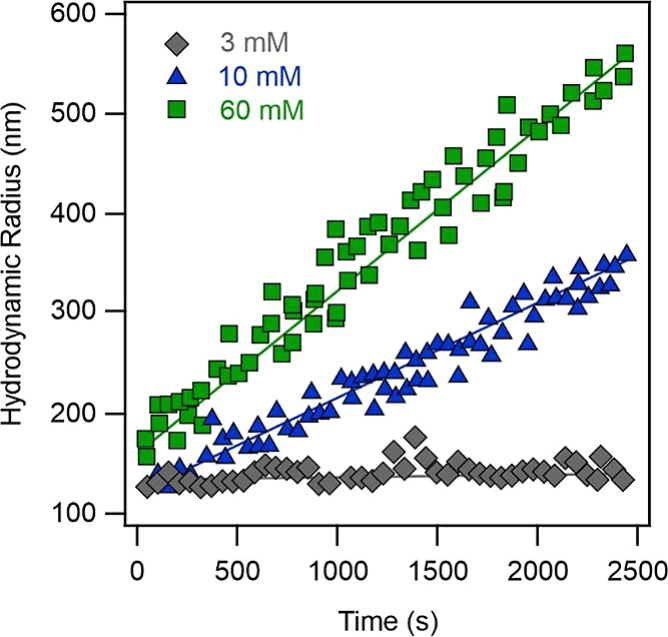
Hydrodynamic radius data measured as a function of time by DLS at different NaCl concentrations and pH 10. The LRD concentration (20 mg/L) was kept constant in the experiments. The solid lines are linear fits used to calculate apparent aggregation rate constant values with eq 4.

The salt-dependent aggregation features were investigated in NaCl solutions. Sodium cation functions as a counterion in the system (opposite sign of charge to the overall charge of LRD), whereas chloride is the co-ion. To assess the ionic strength-dependent colloidal stability of the samples, time-resolved DLS measurements were performed in LRD dispersions at various NaCl concentrations, see examples in Figure 2.

**Figure 3:**
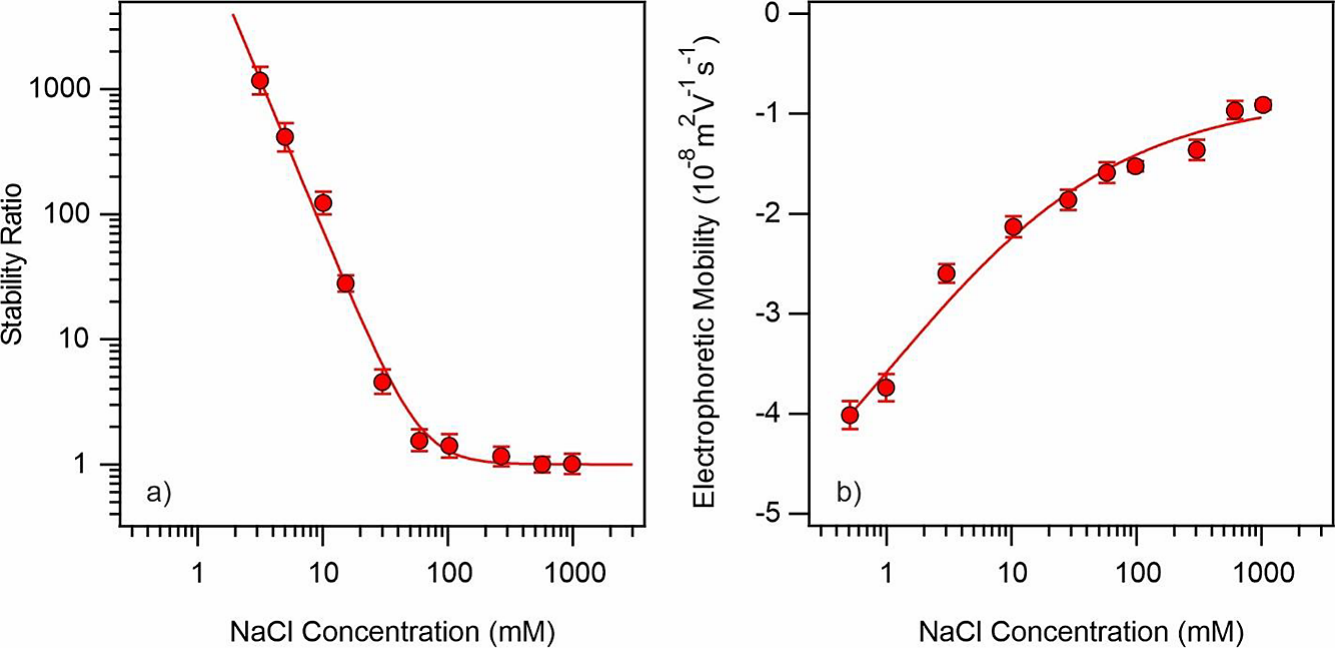
Stability ratio (a) and electrophoretic mobility (b) data of LRD as a function of the NaCl concentration. The solid line in (a) was calculated with eq 7, while it is just to guide the eyes in (b).

The hydrodynamic radius values remained constant within the experimental error at low salt levels (3 mM), while they raised with time by increasing the NaCl concentration (10 and 60 mM). After a threshold value of 60 mM, the slopes of the hydrodynamic radii versus time plots remained constant. The apparent rate constants for LRD aggregation were calculated with eq 4 from data presented in Figure 2. The obtained *k*_DLS_^fast^ values for LRD were (3.8 ± 0.2) × 10^–3^ 1/s. The stability ratio (eq 6) data of LRD were determined at different NaCl concentrations (Figure 3a).

The stability ratios were high at lower ionic strengths, while decreased to one by increasing the NaCl concentration. The CCC of LRD was found to be 60 mM, and this value is comparable to those obtained previously for charged inorganic particles in salt solutions.^8,10,46^ The electrophoretic mobility data decreased as salt concentration increased, which can be attributed to the charge screening effect caused by the dissolved salt constituents (Figure 3b). The mobilities were converted into zeta potentials to obtain σ at the slip plane using eq 3. The resulting value of σ for the LRD in NaCl solutions was −15 mC/m^2^.

These findings unambiguously demonstrate the existence of DLVO-type interparticle forces, which govern the particle aggregation processes. Significant amount of charged groups are present on the particle surface at low salt concentrations, as shown by highly negative mobility values. This leads to the formation of strong EDL forces that stabilize the dispersions. Repulsive forces weaken as a result of the EDL shrinking and screening as the concentration of the added electrolyte rises. Particle aggregation starts, when van der Waals forces surpass the EDL repulsion, and its rate continues to accelerate until it reaches the CCC, beyond which particle aggregation is limited solely by the diffusion.

**Scheme 1:**
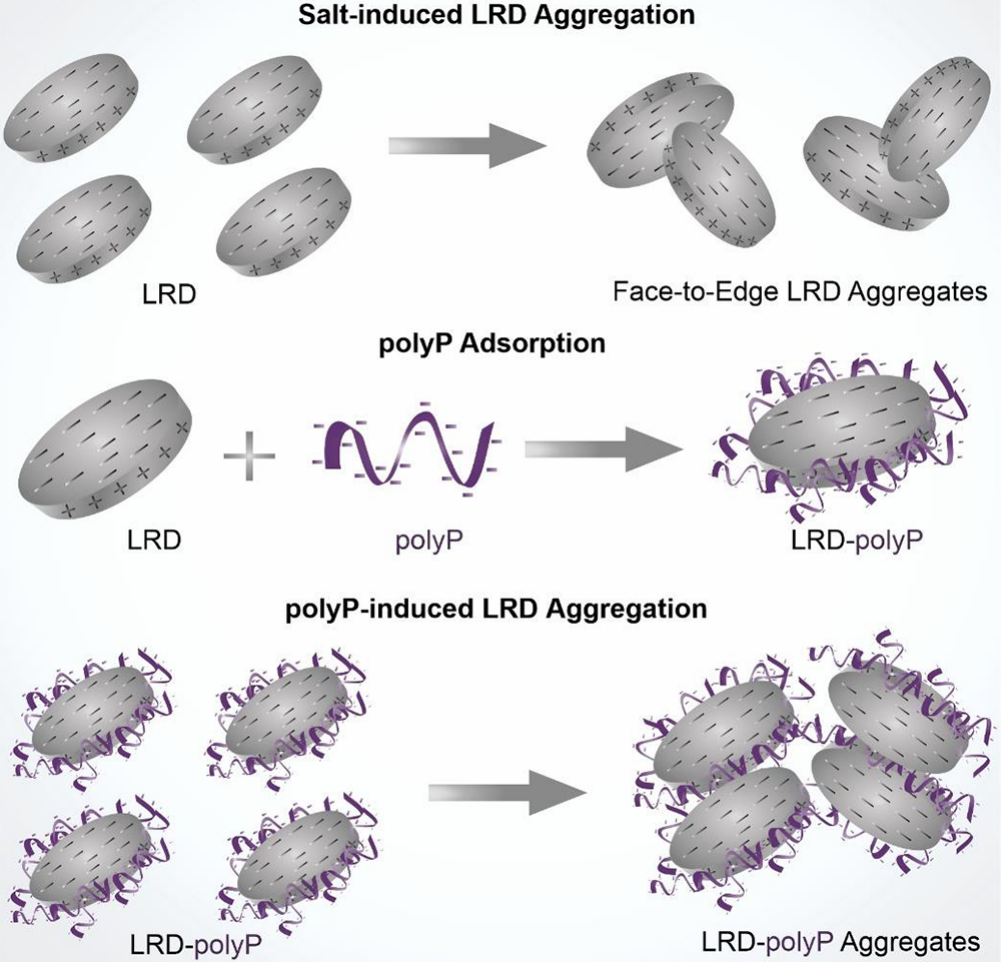
Illustration of the Face-to-Edge Aggregation of LRD (Top), polyP Adsorption on the LRD Edges (Middle), and the Possible Mechanism of polyP-Induced Particle Aggregation through Polymer Bridging Forces (Bottom)

In addition to the above-discussed DLVO-type aggregation mechanism, other scenarios may exist. First, non-DLVO repulsive interactions such as hydration and oscillatory forces^47^ can be present, while their extent is assumed to be smaller compared to EDL repulsion. Face-to-edge aggregation most likely also occur, particularly in the intermediate salt concentration range, in which the LRD charges are not completely screened. In this situation, the positively charged LRD edges are attracted to the oppositely charged faces of the platelets as depicted in Scheme 1 (top).

### Colloidal Stability in the Presence of polyPs

**Figure 4:**
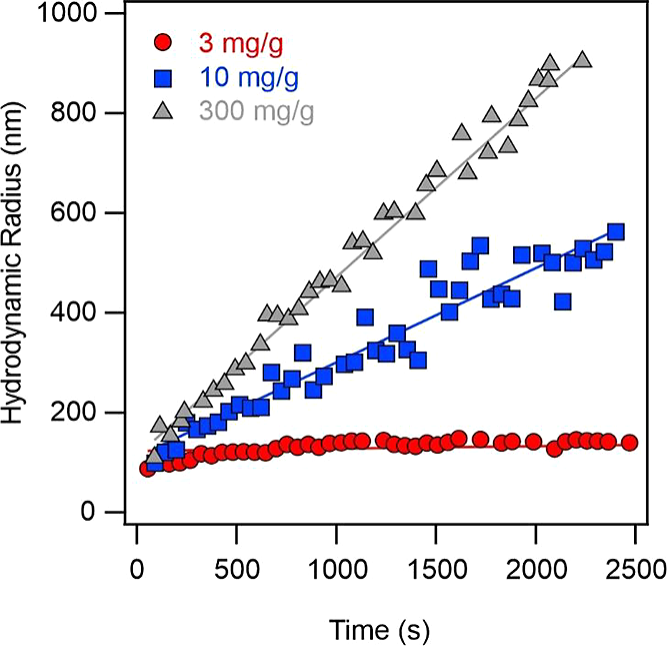
Hydrodynamic radius data measured as a function of time by DLS at different polyP(151) doses at 1 mM ionic strength at pH 10. The solid lines are linear fits used to calculate the apparent aggregation rate constant with eq 4 and subsequently, the stability ratio data with eq 6.

Time-resolved DLS was applied to assess the aggregation stage of LRD in the presence of polyP(103) and polyP(151). At pH 10, both LRD and polyPs are highly charged, while the low hydrodynamic size of the particles allows to perform reliable light scattering measurements. The time-dependent hydrodynamic radii demonstrate that stable samples or slow aggregation processes occur at lower polymer concentrations, while larger polymer loadings led to rapid aggregation of the particles. In Figure 4, representative hydrodynamic radius data as a function of time recorded in the LRD-polyP(151) system are shown.

**Figure 5:**
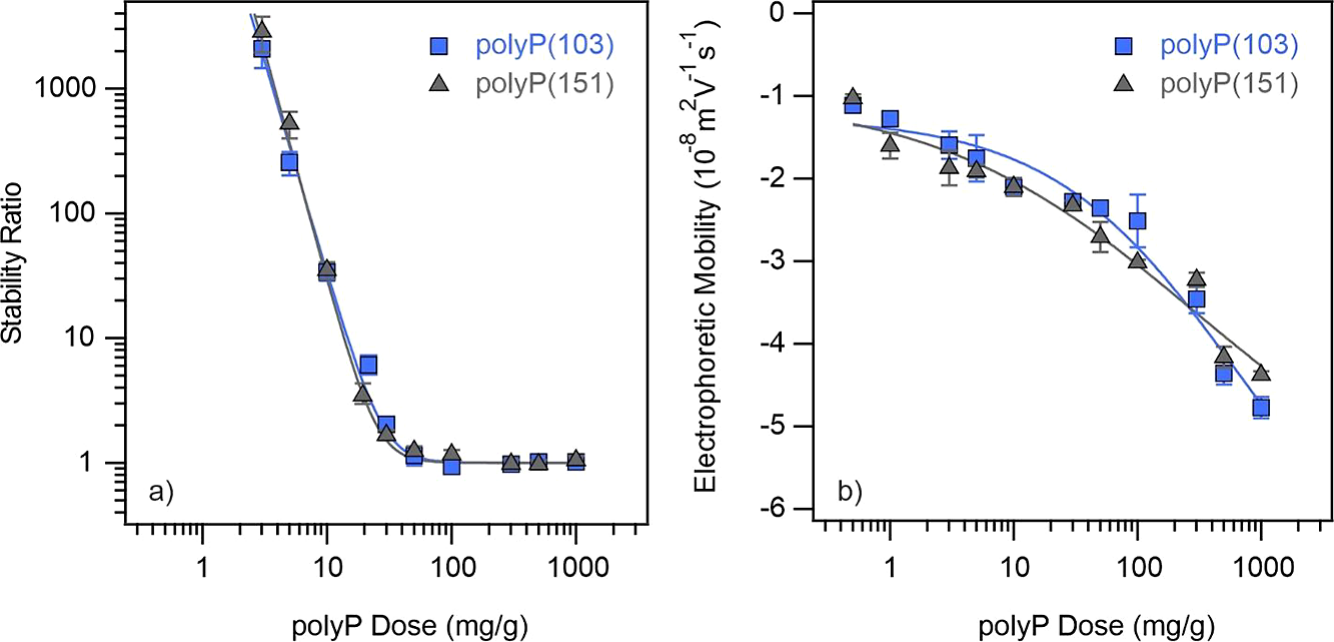
Stability ratio (a) and electrophoretic mobility (b) data of LRD at different polyP doses. The mg/g unit refers to mg polyP per one gram of LRD. The measurements were carried out at 1 mM NaCl concentration as a background electrolyte. The solid line in (a) is the fit by eq 7 (note that polyP dose was used instead of *c*_salt_ in this relation for polymer-containing samples), while it serves to guide the eyes in (b).

Stability ratios were calculated with eq 6, and two main observations can be drawn from the polyP dose-dependent trends (Figure 5a). First, the stability curves were found to be nearly identical for both polyPs within the experimental error range, indicating that the chain length did not influence the rate of aggregation. Second, the data exhibit a pattern similar to that observed in salt solutions (Figure 3a) with distinct regimes of slow and fast aggregation that were clearly separated by a threshold polymer dose. Following the nomenclature used for the CCC, a critical coagulation polyP dose of 27 mg/g was determined by eq 7 for both polyP systems.

Furthermore, the apparent rate constants (see eq 4) obtained in the fast aggregation regimes were found to be (4.0 ± 0.3) × 10^–3^ 1/s and (3.9 ± 0.6) × 10^–3^ 1/s in the presence of polyP(103) and polyP(151), respectively. Accordingly, these values closely resemble the *k*_DLS_^fast^ value obtained at high NaCl concentrations (3.8 ± 0.2) × 10^–3^ 1/s, which implies that the aggregation is diffusion-controlled beyond 27 mg/g polyP dose in both polyP-LRD systems. In other words, although the mechanism or orientation of the LRD platelets in the aggregation may differ, the aggregation kinetics should be very similar regardless of the type of aggregating agents (NaCl or polyP), and LRD diffusion certainly plays a significant role during the process.

Figure 5b shows the electrophoretic mobilities measured under the same experimental conditions as the stability ratios. The values, in general, decreased by increasing the polyP dose and remained negative in the entire concentration range studied. This tendency can be explained by referring to previous studies on LRD-phosphate systems.^22,23^ It was also suggested that the progressive adsorption of polyP on the positively charged edges of LRD (as illustrated in Scheme 1, middle), leads to an increase in the overall negative particle charge at higher polyP doses.^48^ Variations in the degree of polymerization did not yield any significant changes in the electrophoretic mobilities. Specifically, at low polymer dose (1 mg/g polyP dose), almost the same electrophoretic mobility values were measured for the LRD as −(1.27 ± 0.07) × 10^–8^ m^2^/(V s) and −(1.57 ± 0.09) × 10^–8^ m^2^/(V s) in the presence of polyP(103) and polyP(151), respectively. These data decreased to −(4.77 ± 0.12) × 10^–8^ m^2^/(V s) as well as to −(4.37 ± 0.04) × 10^–8^ m^2^/V s at 1000 mg/g polyP dose, indicating the presence of highly charged particles at elevated polyP loadings.

The aforementioned values show that LRD aggregation takes place when the magnitude of charge increases upon polyP adsorption and that the rates rise with the polyP dose. This is in contrast with the prediction of the DLVO theory, which assumes that strong EDL repulsive forces cause stable dispersions of highly charged particles. Therefore, it can be concluded that the interactions responsible for the aggregation of LRD do not originate from the DLVO theory. In other words, van der Waals forces are not sufficient to induce particle aggregation for highly charged particles.^49^ This implies that other forms of attractive interparticle forces will most likely predominate.

First, as an enthalpic attractive interaction, bridging effects between colloidal particles via adsorbed polymer chains have been reported in previous studies.^39,50^ Although at higher solid contents, these effects have also been observed in LRD-polyP systems.^22^ It is highly possible that such an aggregation scenario (as shown in Scheme 1, bottom) occurred in the LRD-polyP dispersions studied. This is because the polyP chain attached to the edges may dangle into the solution phase, making it accessible for another particle with open adsorption sites. It should be noted that this mechanism most likely operates at these intermediate concentration regimes where the edges are not fully saturated with polyP chains.

Second, high concentrations of dissolved polymer chains tend to cause particle aggregation through depletion forces.^51−53^ Accordingly, in particle–polymer dispersions, the presence of nonadsorbing polymers leads to a distinct entropic attraction between the particles. The depletion forces arise due to the difference in the osmotic pressure within the gap between two approaching colloidal particles and the bulk solution. In the case when such a gap is smaller than the size of the polymer, its concentration within the gap will be low resulting in an increase of the attractive force between the particles. In the presented systems at high polyP doses, the edges are entirely covered by polyP chains, and a considerable portion of the polymers may be dissolved in the bulk. As a result, depletion aggregation can be initiated by these chains, where the dissolved polyP molecules act as depletants.

Hence, LRD dispersions can be destabilized by both attractive enthalpic bridging and entropic depletion forces. Furthermore, depending on the experimental conditions, their combination is quite likely to occur in the current LRD-polyP systems. At intermediate polyP doses (about 10–100 mg/g), when polymer chains are able to adsorb to multiple LRD edges, polyP bridging is probably more prominent. On the other hand, at higher polyP doses, depletion attraction becomes predominant where the polymer concentration in bulk solution is sufficiently high to induce aggregation. Studying the balance of these forces at various temperatures and ionic strengths where stability regimes might be found at different polyP doses would be an intriguing follow-up project. In addition, the application of other ionic environments than sodium chloride may also affect the colloidal features of the dispersions. For instance, the presence of different mono and multivalent electrolytes can alter the interparticle forces, as predicted by the Hofmeister series of ions^54^ and the Schulze-Hardy rule,^55^ respectively.

## Conclusions

The primary goal of this study was to evaluate the basic colloidal behavior of commercially available synthetic LRD particles in the presence of two polyPs with different molecular masses. Ionic strength-dependent stability ratio and electrophoretic mobility data followed the trend predicted by the classical DLVO theory, indicating the presence of double-layer repulsion and van der Waals attraction as major interparticle forces. The electrophoretic mobility data of LRD showed a significant increase in magnitude with increasing polyP concentration, which was attributed to polymer adsorption. The consequence of such an adsorption was a decrease in the number of positively charged sites on the edges of the disc-like particles. A continuous increase in the concentration of polyP yielded both slow and fast aggregation regimes for LRD, indicating unstable LRD colloids and the formation of aggregates at higher doses of polyP, as a result of bridging interactions or depletion forces induced by the adsorbed or dissolved polymers, respectively. The degree of polymerization had no discernible impact on the aggregation rate or surface charge since the electrophoretic mobility and stability ratio data of LRD were identical within the experimental error for both polyP samples of different molecular masses. The findings are in good agreement with previously reported results on the aggregation of LRD in the presence of poly- or pyrophosphate, and thus, it is clearly demonstrated that the early stages of particle aggregation govern the dispersion properties of LRD-polyP dispersions. The results revealed that phosphate groups are adsorbed on the LRD disc edges. This adsorption process resulted in particle aggregation, in contrast to other clay types where phosphate derivatives acted as stabilizing agents.

## Supporting Information Available

Results of the potentiometric determination of polyphosphate chains, powder X-ray diffraction analysis, and laser-induced breakdown spectra analysis (PDF)

## References

[ref1] Ruzicka B.; Zaccarelli E. A fresh look at the Laponite phase diagram. Soft Matter 2011, 7, 1268–1286. 10.1039/c0sm00590h.

[ref2] Mongondry P.; Tassin J. F.; Nicolai T. Revised state diagram of Laponite dispersions. J. Colloid Interface Sci. 2005, 283, 397–405. 10.1016/j.jcis.2004.09.043. 15721911

[ref3] Shahin A.; Joshi Y. M. Physicochemical effects in aging aqueous laponite suspensions. Langmuir 2012, 28, 15674–15686. 10.1021/la302544y. 23057660

[ref4] Liu P. F.; Du M. Y.; Clode P.; Yuan P.; Liu J. S.; Leong Y. K. Yield stress and microstructure of composite halloysite-LAPONITE(R) gels: Effects of mixing ratio, surface chemistry, and ageing time. Colloid Surf. A-Physicochem. Eng. Asp. 2022, 640, 128472 10.1016/j.colsurfa.2022.128472.

[ref5] Ruggerone R.; Plummer C. J. G.; Herrera N. N.; Bourgeat-Lami E.; Manson J. A. E. Highly filled polystyrene-laponite nanocomposites prepared by emulsion polymerization. Eur. Polym. J. 2009, 45, 621–629. 10.1016/j.eurpolymj.2008.12.032.

[ref6] Cauvin S.; Colver P. J.; Bon S. A. F. Pickering stabilized miniemulsion polymerization: Preparation of clay armored latexes. Macromolecules 2005, 38, 7887–7889. 10.1021/ma051070z.

[ref7] Mori Y.; Togashi K.; Nakamura K. Colloidal properties of synthetic hectorite clay dispersion measured by dynamic light scattering and small angle X-ray scattering. Adv. Powder Technol. 2001, 12, 45–59. 10.1163/156855201744958.

[ref8] Katana B.; Takács D.; Csapo E.; Szabo T.; Jamnik A.; Szilagyi I. Ion specific effects on the stability of halloysite nanotube colloids-inorganic salts versus ionic liquids. J. Phys. Chem. B 2020, 124, 9757–9765. 10.1021/acs.jpcb.0c07885. 33076658 PMC7660744

[ref9] Leong Y. K. Direct evidence of electric double layer (EDL) repulsive force being responsible for the time-dependent behavior of clay gels in the structural rejuvenation mode. J. Phys. Chem. B 2024, 128, 3784–3793. 10.1021/acs.jpcb.4c00858. 38593457

[ref10] Pavlovic M.; Huber R.; Adok-Sipiczki M.; Nardin C.; Szilagyi I. Ion specific effects on the stability of layered double hydroxide colloids. Soft Matter 2016, 12, 4024–4033. 10.1039/C5SM03023D. 26997621

[ref11] Tomás H.; Alves C. S.; Rodrigues J. Laponite®: A key nanoplatform for biomedical applications?. Nanomed.-Nanotechnol. Biol. Med. 2018, 14, 2407–2420. 10.1016/j.nano.2017.04.016. 28552649

[ref12] Kiaee G.; Dimitrakakis N.; Sharifzadeh S.; Kim H. J.; Avery R. K.; Moghaddam K. M.; Haghniaz R.; Yalcintas E. P.; de Barros N. R.; Karamikamkar S.; Libanori A.; Khademhosseini A.; Khoshakhlagh P. Laponite-based nanomaterials for drug delivery. Adv. Healthc. Mater. 2022, 11, e2102054 10.1002/adhm.202102054. 34990081 PMC8986590

[ref13] Tabesh E.; Kharaziha M.; Mahmoudi M.; Shahnam E.; Rozbahani M. Biological and corrosion evaluation of Laponite®: Poly(caprolactone) nanocomposite coating for biomedical applications. Colloid Surf. A-Physicochem. Eng. Asp. 2019, 583, 123945 10.1016/j.colsurfa.2019.123945.

[ref14] Cummins H. Z. Liquid, glass, gel: The phases of colloidal Laponite. J. Non-Cryst. Solids 2007, 353, 3891–3905. 10.1016/j.jnoncrysol.2007.02.066.

[ref15] Joshi Y. M. Model for cage formation in colloidal suspension of laponite. J. Chem. Phys. 2007, 127, 081102 10.1063/1.2779026. 17764222

[ref16] Au P. I.; Hassan S.; Liu J. S.; Leong Y. K. Behaviour of laponite gels: Rheology, ageing, pH effect and phase state in the presence of dispersant. Chem. Eng. Res. Des. 2015, 101, 65–73. 10.1016/j.cherd.2015.07.023.

[ref17] Brunchi C.-E.; Morariu S. Laponite® - From dispersion to gel - Structure, properties, and applications. Molecules 2024, 29, 2823 10.3390/molecules29122823. 38930887 PMC11206873

[ref18] Bhattacharjee S.; Singh B. P.; Besra L.; Sengupta D. K. Performance evaluation of dispersants through streaming potential measurements. J. Dispersion Sci. Technol. 2005, 26, 365–370. 10.1081/DIS-200049616.

[ref19] Farrokhpay S.; Morris G. E.; Britcher L. G. Stability of sodium polyphosphate dispersants in mineral processing applications. Miner. Eng. 2012, 39, 39–44. 10.1016/j.mineng.2012.07.001.

[ref20] Saracini J.; de Assis I. C. M.; Peiter G. C.; Busso C.; de Oliveira R. J.; Felix J. F.; Bini R. A.; Schneider R. Borophosphate glasses as active agents for antimicrobial hydrogels. Int. J. Pharm. 2023, 644, 123323 10.1016/j.ijpharm.2023.123323. 37597596

[ref21] Castellini E.; Berthold C.; Malferrari D.; Bemini F. Sodium hexametaphosphate interaction with 2:1 clay minerals Illite and montmorillonite. Appl. Clay Sci. 2013, 83–84, 162–170. 10.1016/j.clay.2013.08.031.

[ref22] Motta R. J. B.; Almeida A. Z. F.; de Lima B. L. B.; Schneider R.; Balaban R. D.; van Duijneveldt J. S.; de Oliveira R. J. Polyphosphates can stabilize but also aggregate colloids. Phys. Chem. Chem. Phys. 2020, 22, 15–19. 10.1039/C9CP05225A. 31815261

[ref23] Mongondry P.; Nicolai T.; Tassin J. F. Influence of pyrophosphate or polyethylene oxide on the aggregation and gelation of aqueous laponite dispersions. J. Colloid Interface Sci. 2004, 275, 191–196. 10.1016/j.jcis.2004.01.037. 15158398

[ref24] Bujok S.; Konefal M.; Konefal R.; Nevoralová M.; Bednarz S.; Mielczarek K.; Benes H. Insight into the aqueous Laponite® nanodispersions for self-assembled poly(itaconic acid) nanocomposite hydrogels: The effect of multivalent phosphate dispersants. J. Colloid Interface Sci. 2022, 610, 1–12. 10.1016/j.jcis.2021.12.055. 34922067

[ref25] Ordikhani F.; Dehghani M.; Simchi A. Antibiotic-loaded chitosan-Laponite films for local drug delivery by titanium implants: cell proliferation and drug release studies. J. Mater. Sci. Mater. Med. 2015, 26, 269 10.1007/s10856-015-5606-0. 26507202

[ref26] Kafili G.; Tamjid E.; Niknejad H.; Simchi A. Development of injectable hydrogels based on human amniotic membrane and polyethyleneglycol-modified nanosilicates for tissue engineering applications. Eur. Polym. J. 2022, 179, 111566 10.1016/j.eurpolymj.2022.111566.

[ref27] Jatav S.; Joshi Y. M. Chemical stability of Laponite in aqueous media. Appl. Clay Sci. 2014, 97–98, 72–77. 10.1016/j.clay.2014.06.004.

[ref28] Shan D.; Cosnier S.; Mousty C. Layered double hydroxides: An attractive material for electrochemical biosensor design. Anal. Chem. 2003, 75, 3872–3879. 10.1021/ac030030v. 14572056

[ref29] Suterio N.; Bazzo G. C.; Rauber G. S.; Silva A. H.; Caon T.; Parize A. L.; Creczynski-Pasa T. B.; Stulzer H. K. Laponite® gel formulation containing simvastatin for melanoma treatment. Appl. Clay Sci. 2022, 228, 106651 10.1016/j.clay.2022.106651.

[ref30] Christ J. J.; Willbold S.; Blank L. M. Methods for the analysis of polyphosphate in the life sciences. Anal. Chem. 2020, 92, 4167–4176. 10.1021/acs.analchem.9b05144. 32039586

[ref31] Momeni A.; Filiaggi M. J. Synthesis and characterization of different chain length sodium polyphosphates. J. Non-Cryst. Solids 2013, 382, 11–17. 10.1016/j.jnoncrysol.2013.10.003.

[ref32] Kramida A.; Ralchenko Y.; Reader J.; NIST ASD Team. NIST atomic spectra database, 5.11 ed.; National Institute of Standards and Technology: Gaithersburg, MD, USA, 2023.

[ref33] Delgado A. V.; Gonzalez-Caballero E.; Hunter R. J.; Koopal L. K.; Lyklema J. Measurement and interpretation of electrokinetic phenomena - (IUPAC technical report). Pure Appl. Chem. 2005, 77, 1753–1805. 10.1351/pac200577101753. 17368660

[ref34] Evans D. F.; Wennerstrom H. The colloidal domain; John Wiley, 1999.

[ref35] Trefalt G.; Szilagyi I.; Borkovec M. Poisson-Boltzmann description of interaction forces and aggregation rates involving charged colloidal particles in asymmetric electrolytes. J. Colloid Interface Sci. 2013, 406, 111–120. 10.1016/j.jcis.2013.05.071. 23827478

[ref36] Holthoff H.; Egelhaaf S. U.; Borkovec M.; Schurtenberger P.; Sticher H. Coagulation rate measurements of colloidal particles by simultaneous static and dynamic light scattering. Langmuir 1996, 12, 5541–5549. 10.1021/la960326e.

[ref37] Schneider C.; Hanisch M.; Wedel B.; Jusufi A.; Ballauff M. Experimental study of electrostatically stabilized colloidal particles: Colloidal stability and charge reversal. J. Colloid Interface Sci. 2011, 358, 62–67. 10.1016/j.jcis.2011.02.039. 21419414

[ref38] Hassan P. A.; Rana S.; Verma G. Making sense of Brownian motion: Colloid characterization by dynamic light scattering. Langmuir 2015, 31, 3–12. 10.1021/la501789z. 25050712

[ref39] Iselau F.; Xuan T. P.; Trefalt G.; Matic A.; Holmberg K.; Bordes R. Formation and relaxation kinetics of starch-particle complexes. Soft Matter 2016, 12, 9509–9519. 10.1039/C6SM01312K. 27853795

[ref40] Grolimund D.; Elimelech M.; Borkovec M. Aggregation and deposition kinetics of mobile colloidal particles in natural porous media. Colloid Surf. A 2001, 191, 179–188. 10.1016/S0927-7757(01)00773-7.

[ref41] Gustavson K. H.; Larsson A. The interaction of polymetaphosphates with hide protein. Acta Chem. Scand. 1951, 5, 1221–1243. 10.3891/acta.chem.scand.05-1221.

[ref42] Kaser S.; Guérineau T.; Strutynski C.; Zaki R.; Dussauze M.; Durand E.; Messaddeq S. H.; Danto S.; Messaddeq Y.; Cardinal T. Novel optical amorphous phosphate materials with a low melting temperature. Mater. Adv. 2022, 3, 4600–4607. 10.1039/D1MA00995H.

[ref43] Parks G. A. The isoelectric points of solid oxides, solid hydroxides, and aqueous hydroxo complex systems. Chem. Rev. 1965, 65, 177–198. 10.1021/cr60234a002.

[ref44] Lee H. M.; Kim Y. W.; Go E. M.; Revadekar C.; Choi K. H.; Cho Y.; Kwak S. K.; Park B. J. Direct measurements of the colloidal Debye force. Nat. Commun. 2023, 14, 3838 10.1038/s41467-023-39561-8. 37380657 PMC10307906

[ref45] Du M. Y.; Liu J. S.; Clode P. L.; Leong Y. K. Surface chemistry, rheology and microstructure of purified natural and synthetic hectorite suspensions. Phys. Chem. Chem. Phys. 2018, 20, 19221–19233. 10.1039/C8CP01382A. 29987309

[ref46] Hegedus T.; Takács D.; Vasarhelyi L.; Szilagyi I.; Konya Z. Specific ion effects on aggregation and charging properties of boron nitride nanospheres. Langmuir 2021, 37, 2466–2475. 10.1021/acs.langmuir.0c03533. 33555897 PMC8023703

[ref47] Zachariah Z.; Heuberger M. P.; Espinosa-Marzal R. M. Colloidal interactions-DLVO theory and beyond. In One Hundred Years of Colloid Symposia: Looking Back and Looking Forward; ACS Symposium Series; American Chemical Society, 2023; Vol. 1457, pp 31–47.

[ref48] Shang C.; Rice J. A. Invalidity of deriving interparticle distance in clay-water systems using the experimental structure factor maximum obtained by small-angle scattering. J. Colloid Interface Sci. 2005, 283, 94–101. 10.1016/j.jcis.2004.06.032. 15694428

[ref49] Galli M.; Saringer S.; Szilagyi I.; Trefalt G. A simple method to determine critical coagulation concentration from electrophoretic mobility. Colloid Interfac. 2020, 4, 20 10.3390/colloids4020020.

[ref50] Brunel F.; Pochard I.; Gauffine S.; Turesson M.; Labbez C. Structure and yielding of colloidal silica gels varying the range of interparticle interactions. J. Phys. Chem. B 2016, 120, 5777–5785. 10.1021/acs.jpcb.6b04047. 27284941

[ref51] Feng L.; Laderman B.; Sacanna S.; Chaikin P. Re-entrant solidification in polymer-colloid mixtures as a consequence of competing entropic and enthalpic attractions. Nat. Mater. 2015, 14, 61–65. 10.1038/nmat4109. 25326826

[ref52] Huang H.; Ruckenstein E. Effect of steric double-layer and depletion interactions on the stability of colloids in systems containing a polymer and an electrolyte. Langmuir 2006, 22, 4541–4546. 10.1021/la0602057. 16649761

[ref53] Luan L. Y.; Li W.; Liu S. Y.; Sun D. J. Phase behavior of mixtures of positively charged colloidal platelets and nonadsorbing polymer. Langmuir 2009, 25, 6349–6356. 10.1021/la804023b. 19466785

[ref54] Oncsik T.; Trefalt G.; Borkovec M.; Szilagyi I. Specific ion effects on particle aggregation induced by monovalent salts within the Hofmeister series. Langmuir 2015, 31, 3799–3807. 10.1021/acs.langmuir.5b00225. 25777544

[ref55] Trefalt G.; Szilagyi I.; Borkovec M. Schulze-Hardy rule revisited. Colloid Polym. Sci. 2020, 298, 961–967. 10.1007/s00396-020-04665-w.

