# Supplementary material for: The Impact of Polyphosphates on the Colloidal Stability of Laponite Particles
Source: J Phys Chem B. 2024 Jul 9;128(28):6957–65. doi: 10.1021/acs.jpcb.4c03193 (PMC11264265; doi:10.1021/acs.jpcb.4c03193)
Supplement: Supplementary file 1 — jp4c03193_si_001.pdf [file jp4c03193_si_001.pdf]

Supporting Information (SI)

The Impact of Polyphosphates on the Colloidal Stability of Laponite Particles

Bojana Katana[a], João Baptista[b,c], Ricardo Schneider[b], Rodrigo José de Oliveira[d], and István Szilágyi[a]**

[a]MTA-SZTE Momentum Biocolloids Research Group, Department of Physical Chemistry and Materials Science, Interdisciplinary Centre of Excellence, University of Szeged, 6720 Szeged, Hungary

[b]Group of Polymers and Nanostructures, Federal Technological University of Paraná – UTFPR, 85902-490, Toledo, Paraná, Brazil

[c]University of São Paulo – USP, Chemical Engineering, 05508-800, São Paulo, Brazil

[d]Physical Chemistry of Materials Group, State University of Paraíba – UEPB, 58429-500 Campina Grande, Paraíba, Brazil

*Corresponding Authors:

Email: szistvan@chem.u-szeged.hu (I.S.)

Email: deoliveirarj@servidor.uepb.edu.br (R.J.O.)

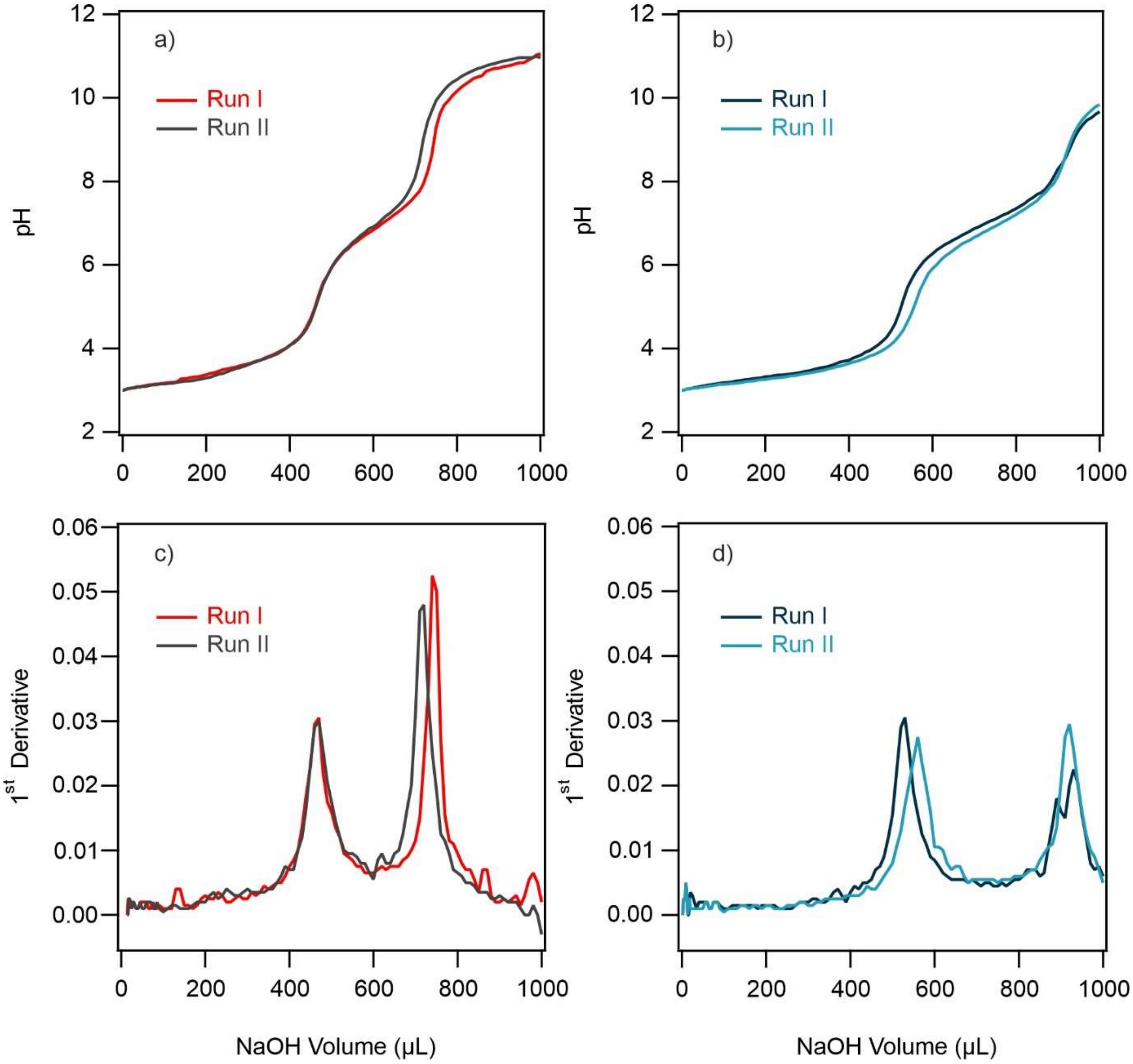

Figure S1. Determination of polyP chain size by a potentiometric titration method. The pH values as a function of the volume of 0.1 M NaOH at a rate of 7 °C/min (a) and, 20 °C/min (b), and first derivative of the titration curves at a rate of 7 °C/min (c) and, 20 °C/min (d).

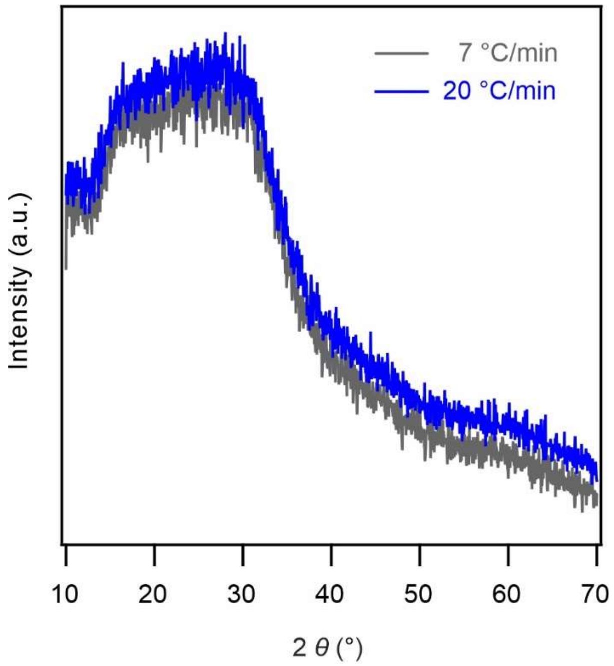

Figure S2. Powder X-ray diffraction analysis of phosphate-based glasses obtained at heating rates of 7 °C/min (polyP151, gray line) and 20 °C/min (polyP103, blue line).

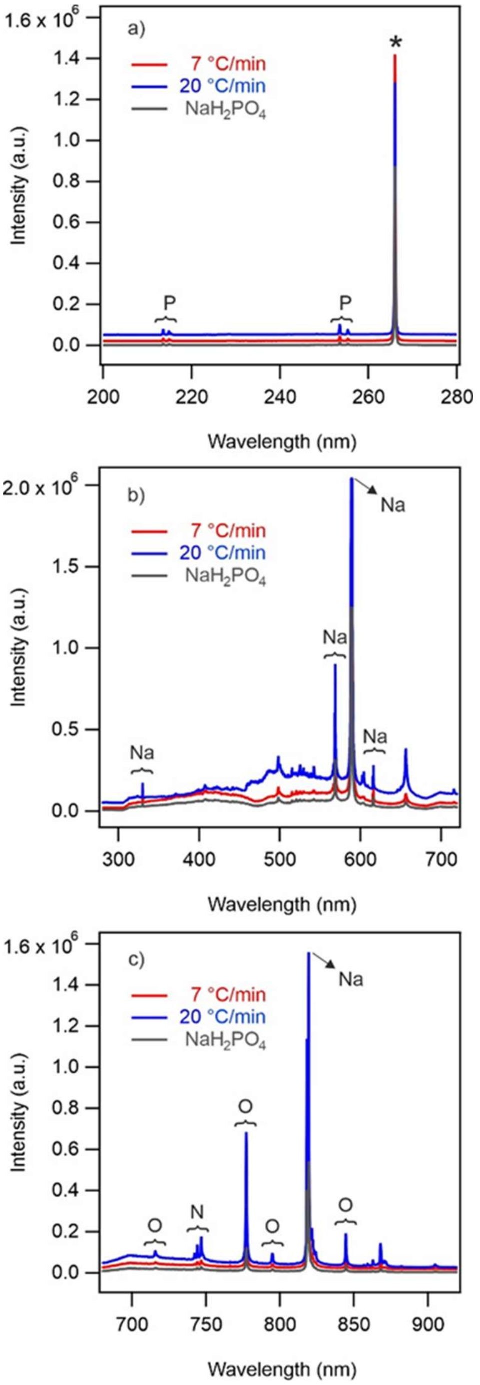

Figure S3. LIBS analysis for phosphate-based glasses obtained at different heating rates (7 °C/min (polyP151, red line), 20 °C/min (polyP103, blue line), and NaH_2PO_4 raw chemical (gray line)). The symbol (*) denotes the laser line at 266 nm. The graphs show the spectra at different ranges, such as (a) 200-280 nm, (b) 280-670 nm, and (c) 670-850 nm.
